# Newly Formed Reticulated Platelets Undermine Pharmacokinetically Short-Lived Antiplatelet Therapies

**DOI:** 10.1161/ATVBAHA.116.308763

**Published:** 2017-03-02

**Authors:** Paul C. Armstrong, Thomas Hoefer, Rebecca B. Knowles, Arthur T. Tucker, Melissa A. Hayman, Plinio M. Ferreira, Melissa V. Chan, Timothy D. Warner

**Affiliations:** From The William Harvey Research Institute, Barts & the London School of Medicine & Dentistry, Queen Mary University of London, Charterhouse Square, London, United Kingdom.

**Keywords:** aspirin, blood platelets, platelet aggregation, thienopyridines, thrombosis

## Abstract

**Objective—:**

Aspirin together with thienopyridine P2Y_12_ inhibitors, commonly clopidogrel, is a cornerstone of antiplatelet therapy. However, many patients receiving this therapy display high on-treatment platelet reactivity, which is a major therapeutic hurdle to the prevention of recurrent thrombotic events. The emergence of uninhibited platelets after thrombopoiesis has been proposed as a contributing factor to high on-treatment platelet reactivity. Here, we investigate the influences of platelet turnover on platelet aggregation in the face of different dual-antiplatelet therapy strategies.

**Approach and Results—:**

Traditional light transmission aggregometry, cytometry, advanced flow cytometric imaging, and confocal microscopy were used to follow the interactions of populations of platelets from healthy volunteers and patients with stable cardiovascular disease. Newly formed, reticulated platelets overproportionately contributed to, and clustered at, the core of forming aggregates. This phenomenon was particularly observed in samples from patients treated with aspirin plus a thienopyridine, but was absent in samples taken from patients treated with aspirin plus ticagrelor.

**Conclusions—:**

Reticulated platelets are more reactive than older platelets and act as seeds for the formation of platelet aggregates even in the presence of antiplatelet therapy. This is coherent with the emergence of an uninhibited subpopulation of reticulated platelets during treatment with aspirin plus thienopyridine, explained by the short pharmacokinetic half-lives of these drugs. This phenomenon is absent during treatment with ticagrelor, because of its longer half-life and ability to act as a circulating inhibitor. These data highlight the important influences of pharmacokinetics on antiplatelet drug efficacies, especially in diseases associated with increased platelet turnover.

## Abstract 2

Supplemental Digital Content is available in the text.

Platelets are central to the processes underlying atherothrombotic events and consequently are the target of well-established prophylactic therapy. The drug dosing regimen referred to as dual-antiplatelet therapy (DAPT) typically comprises aspirin combined with a P2Y_12_ receptor antagonist, commonly the thienopyridine compound clopidogrel.^1–4^ The reoccurrence of thrombotic events during therapy represents a major therapeutic hurdle and is associated with high on-treatment platelet reactivity (HTPR).^3,4^ However, the causes of HTPR and thrombotic complications are complex and require deeper investigation to improve antithrombotic therapies.^3–5^

One potential contributing factor to HTPR is an increased rate of platelet turnover. There are a notable number of pathological states linked to HTPR where platelet turnover and the circulating levels of newly formed immature platelets are increased. In particular, a recent study has associated elevated immature platelet counts, a measure of platelet turnover, with adverse cardiovascular outcomes.^6^ This relationship is clearly demonstrated in patients with chronic renal failure requiring hemodialysis whose reticulated platelet proportion increases 3-fold.^7^ In addition, there is a strong association between poor clopidogrel responsiveness and increased thrombotic risk.^8,9^ Nonetheless, a pathophysiological mechanism has yet to be identified.

Aspirin and clopidogrel, the most widely used P2Y_12_ receptor antagonist, irreversibly bind their respective targets but are short-lived in the circulation. This suggests that as standard daily doses of either drug are quickly cleared newly formed platelets subsequently entering the circulation will remain uninhibited until the next dose is taken. We have recently demonstrated that uninhibited platelets can act as seeds for aggregate formation during antiplatelet therapy.^10^ Therefore, in patients with pathologies in which platelet production is increased larger subpopulations of these uninhibited platelets will arise. Compounding this, newly produced immature, or reticulated, platelets seem to be generally more reactive.^11^

The more recently developed nonthienopyridine P2Y_12_ antagonist ticagrelor is, unlike thienopyridines, pharmacokinetically long-lived with a circulating half-life of ≈8 hours. With standard twice daily dosing, it consistently circulates at inhibitory concentrations. Also unlike thienopyridines, ticagrelor acts directly as a reversibly binding antagonist of P2Y_12_ receptors and may consequently provide more thorough antithrombotic cover than short-lived thienopyridines.^12^

Here, we compare pharmacokinetically different antithrombotic regimens in healthy volunteers and examine their relationship with uninhibited platelets. In a previous in vitro study, we mimicked the in vivo interaction of differently inhibited platelet populations by separately labeling and recombining platelets.^10^ In this ex vivo study, we have directly examined the functionality of reticulated platelets, those most likely to be uninhibited, in samples from patients with stable cardiovascular disease and receiving DAPT. Finally, we describe a mechanism by which newly formed reticulated platelets may promote HTPR and potentially explain the reported increased effectiveness of ticagrelor over thienopyridines.^13,14^

## Materials and Methods

Materials and Methods are available in the online-only Data Supplement.

## Results

### In Vitro Modeling Identifies Differential Inhibition Between Thienopyridines and Ticagrelor

**Figure 1.**
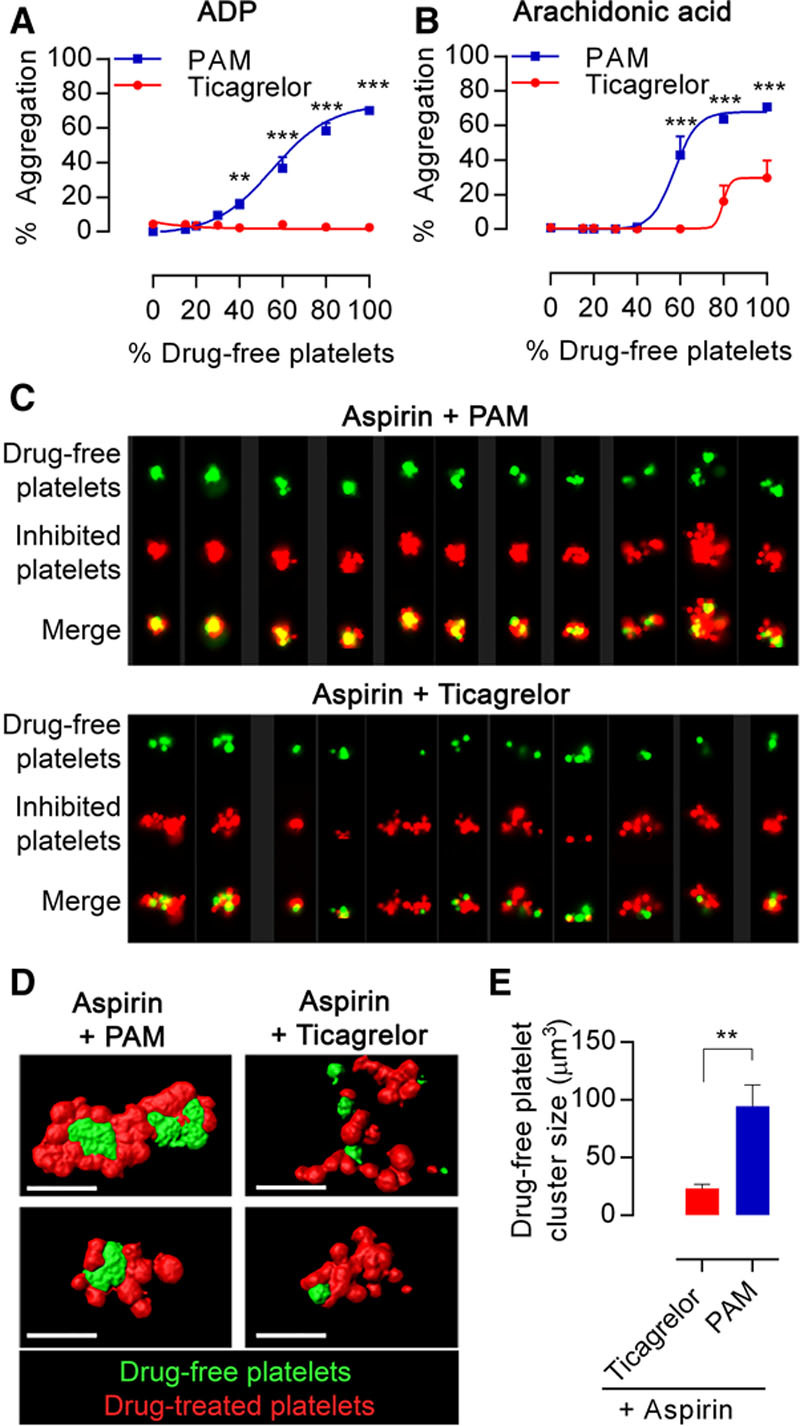
Ticagrelor reduces aggregation and prevents formation of drug-free platelet cores during aggregate formation in vitro. Platelet-rich plasma (PRP) derived from blood preincubated with aspirin (30 μmol/L) and prasugrel active metabolite (PAM; 3 μmol/L) or ticagrelor (1.35 μmol/L) was mixed in a range of proportions with PRP from blood preincubated with respective vehicles or with ticagrelor (1.35 μmol/L) to reflect mid-dose *t*=6-h levels. Aggregation in response to (**A**) ADP 20 μmol/L or (**B**) arachidonic acid 1 mmol/L was determined by light transmission aggregometry. Data presented as mean±SEM and compared by 2-way ANOVA (n=4, **P<0.01, ***P<0.001). **C**, Multiple images captured by ImageStreamX of aggregates (mixtures of 85% aspirin+PAM-pretreated platelets or aspirin+ticagrelor pretreated platelets plus 15% uninhibited platelets). Each panel contains columns with following image sets: drug-free (green), inhibited platelets (red), merged image. **D**, Representative confocal images of aggregates (**left**) formed from mixtures comprising 85% aspirin+PAM-pretreated platelets or aspirin+ticagrelor-pretreated (green) and 15% uninhibited platelets (red). **E**, Images were analyzed for size of the uninhibited platelet particles. Data are presented as mean±SEM and compared by *t* test (n=4; **P<0.01).

Drug-free platelets were added to platelets preincubated with drug to model the in vitro functional consequences of platelet turnover during DAPT treatment comprising aspirin+prasugrel or aspirin+ticagrelor. This modeling demonstrated that for samples treated with prasugrel active metabolite (PAM) responses to ADP returned with increases in the proportion of inhibitor-free platelets. In contrast, aggregatory responses remained inhibited in samples treated with ticagrelor (Figure 1A and 1B). Analysis by flow cytometry and confocal microscopy of aggregates formed from labeled platelet subpopulations indicated that drug-free platelets were overproportionately recruited to the formed aggregates in samples treated with aspirin+PAM. This was evidenced by clear clustering of uninhibited platelets at the cores of the formed aggregates. In contrast, analysis of samples treated with aspirin+ticagrelor did not show this bias (Figure 1C). Quantitative analyses of aggregate images obtained by confocal microscopy demonstrated a significantly bigger core volume when drug-free platelets were mixed with aspirin+PAM–treated samples (95±18 μm^3^) than with aspirin+ticagrelor–treated samples (24±4 μm^3^; Figure 1D and 1E).

### Ticagrelor but Not Prasugrel Therapy Inhibits Drug-Free Platelets on In Vitro Transfusion

**Figure 2.**
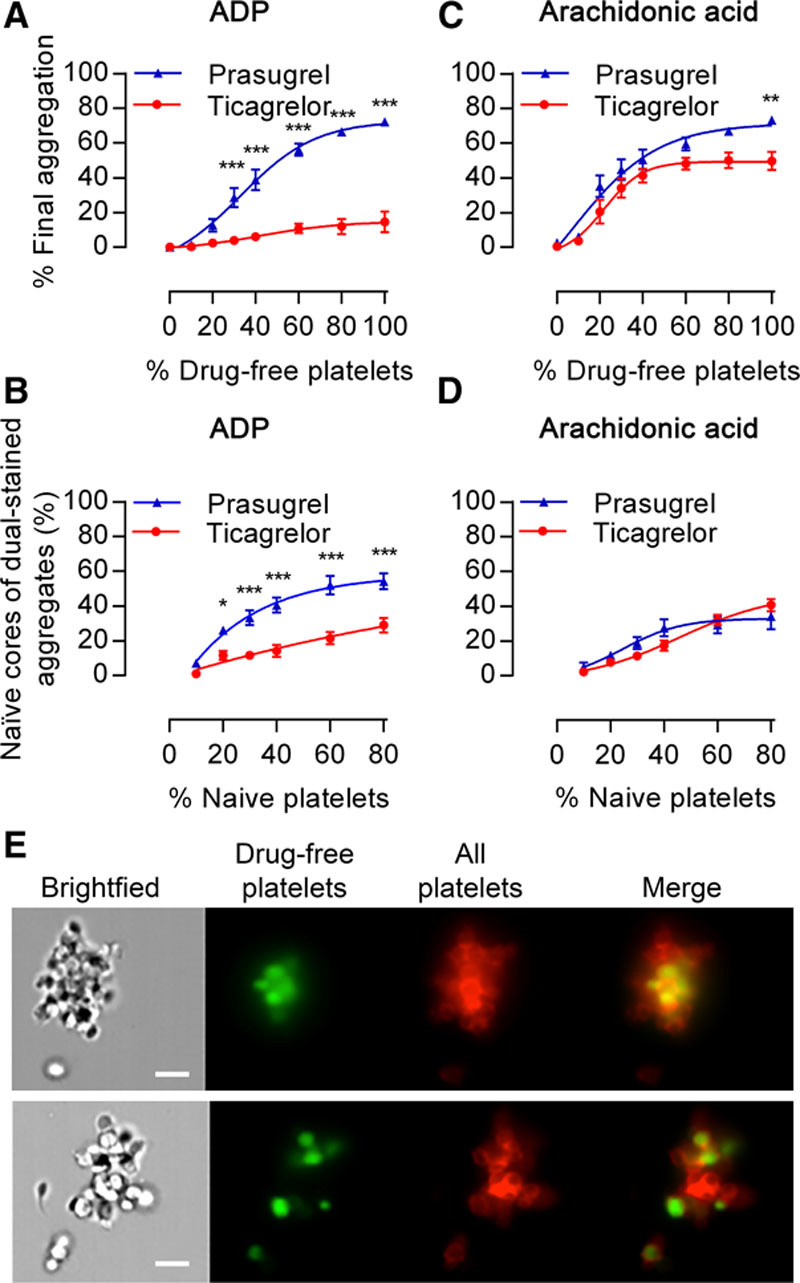
Drug-free platelets restore ex vivo aggregation responses differentially in the presence of prasugrel or ticagrelor. Platelet-rich plasma (PRP) isolated from individuals 6 h after receiving aspirin+prasugrel or aspirin+ticagrelor was mixed with increasing proportions of drug-free platelets and then stimulated. Final aggregation of samples stimulated with (**A**) ADP (20 μmol/L) or (**C**) arachidonic acid (1 mmol/L). **B** and **D**, Aggregates (%) where cores comprise naive platelets were blind scored and calculated from flow cytometric images of platelet aggregates from corresponding samples. One hundred four aggregates assessed per individual sample, with data presented as mean±SEM and compared by 2-way ANOVA (n=10 samples; *P<0.05, **P<0.01, ***P<0.001). **E**, Representative flow cytometric imaging (×60 objective) of aggregates formed in response to ADP (20 μmol/L) from 80%:20% mixtures of aspirin+prasugrel–inhibited platelet-rich plasma (PRP) or aspirin+ticagrelor-inhibited PRP obtained 6 h after the last drug dose was administered (red) and drug-free platelets (green). Scale bars, 7 μm.

Having identified pharmacological differences between PAM and ticagrelor in vitro, we tested differences between prasugrel and ticagrelor in their ability to inhibit drug-free platelets in a larger inhibited environment using an ex vivo approach in healthy volunteers who had either taken aspirin+prasugrel or aspirin+ticagrelor for 7 days. Blood was collected at estimated peak concentrations of PAM (30 minutes after last dose) and ticagrelor (120 minutes after last dose), as well as at 6 hours after the last dose, and platelet-rich plasma (PRP) was made. Drug-free platelets from healthy volunteers were then combined with PRP collected from treated subjects to model various rates of platelet turnover, ranging from no turnover (*x*=0%) to full turnover of new platelets (*x*=100%), and aggregation responses were determined. In samples derived from volunteers treated with aspirin+prasugrel, responses to ADP recovered as the proportion of drug-free platelets was increased, whereas responses remained strongly inhibited in samples from volunteers who had received aspirin+ticagrelor (Figure 2A; Figure II in the online-only Data Supplement). In aspirin+ticagrelor samples, the addition of naive drug-free platelets also produced a smaller increase in the response to arachidonic acid (AA) than in aspirin+prasugrel samples (Figure 2C; Figure II in the online-only Data Supplement).

**Figure 3.**
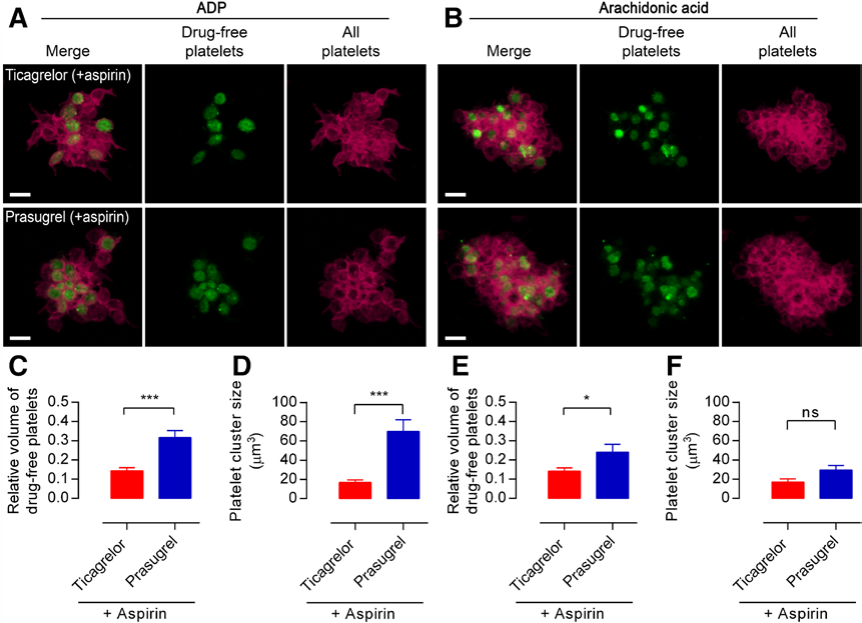
Drug-free platelets form cores within aggregates in the presence of prasugrel but not of ticagrelor. Representative confocal images of (**A**) ADP-stimulated or (**B**) arachidonic acid–stimulated aggregates formed from 80%:20% mixtures of aspirin+prasugrel–inhibited platelet-rich plasma (PRP) or aspirin+ticagrelor–inhibited PRP obtained 6 h after the last drug dose was administered (red) and drug-free platelets (green), conditions as in Figure 2. Images were analyzed for (**C** and **E**) volume of the drug-free platelet particles relative to the total aggregate volume and (**D** and **F**) average size of drug-free platelet clusters. Scale bars, 5 μm. Data are presented as mean±SEM and compared by *t* test (n=7–10; *P<0.05, ***P<0.001).

After light transmission aggregometry, PRP underwent high-throughput flow cytometric imaging to assess aggregate structures. Aggregates (mean=104 per individual sample) were blindly assessed for the proportion of drug-free core aggregates relative to total aggregates (Figures 2E; Figure II in the online-only Data Supplement). After stimulation by ADP, aggregates formed in PRP derived from volunteers treated with aspirin+prasugrel contained a higher proportion of drug-free cores than those formed in PRP derived from volunteers treated with aspirin+ticagrelor (Figure 2B). In contrast, no such difference in proportion was observed between volunteer groups for aggregates formed after AA stimulation (Figure 2D). Further confocal imaging of aggregates from populations comprising 20% drug-free and 80% inhibited platelets confirmed in response to ADP the formation of platelet aggregates with a core of drug-free platelets in aspirin+prasugrel–treated samples, but not in aspirin+ticagrelor–treated samples (Figure 3A). Moreover, quantitative analysis of these images demonstrated fewer drug-free platelets were recruited (relative volumes 0.14±0.02 versus 0.32±0.04 μm^3^, respectively; *P*<0.001; Figure 3C) and smaller drug-free cores were formed (17±3 versus 70±12 μm^3^; *P*<0.001; Figure 3D) in aspirin+ticagrelor–treated samples compared with aspirin+prasugrel–treated samples. Differences in distribution of platelet subpopulations after stimulation by AA were less pronounced (Figure 3B, 3E, and 3F) but were similarly observed at plasma peak time-points (Figure III in the online-only Data Supplement).

### Reticulated Platelets Are More Reactive Than Older Platelets and Locate to the Core of Aggregates

**Figure 4.**
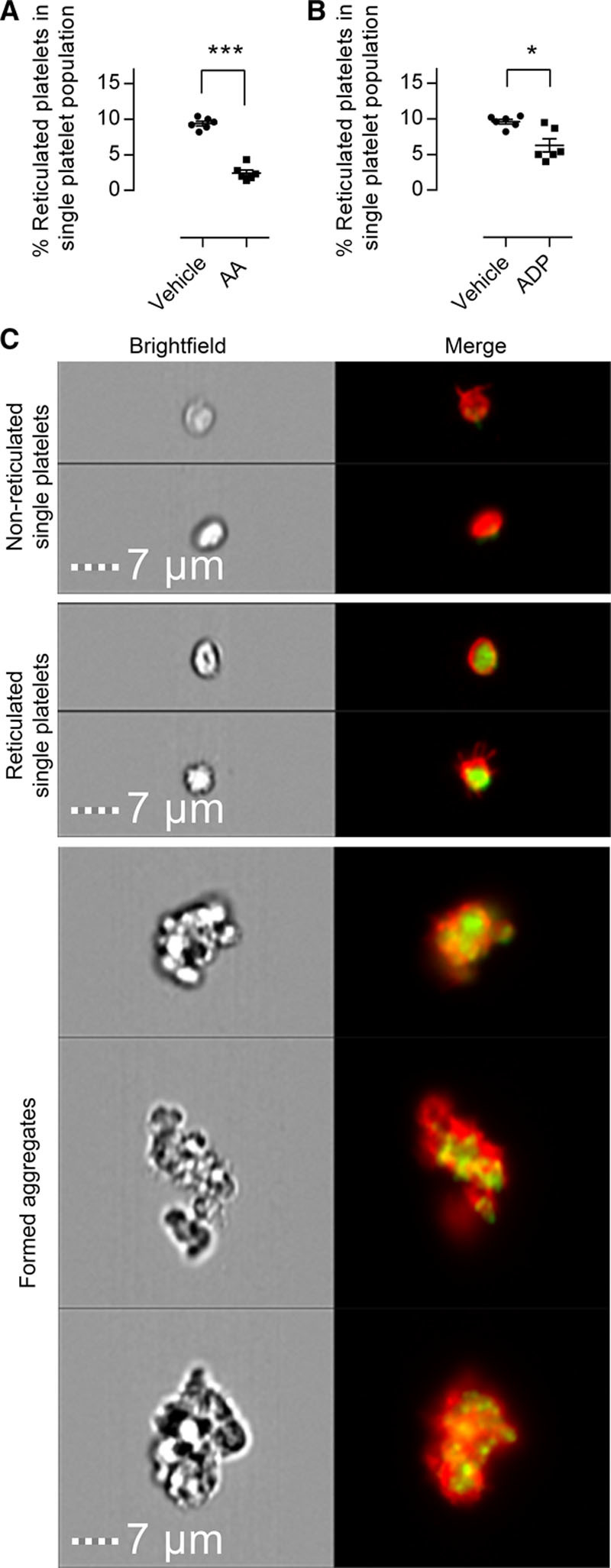
Reticulated platelets display elevated reactivity in response to both arachidonic acid (AA) and ADP. The proportion of reticulated platelets among nonaggregated platelets was assessed by flow cytometry in platelet-rich plasma incubated with vehicle, (**A**) AA, or (**B**) ADP. **C**, Representative flow cytometric images of nonreticulated and reticulated (mRNA stain green) single platelets (red), as well as aggregates formed in response to ADP. Scale bars, 7 μm. Data presented as individual data points with overlaid mean±SEM and compared by paired *t* test (n=6; *P<0.05, ***P<0.001).

To monitor the reactivity of newly formed platelets, also called reticulated platelets because of the presence of mRNA, platelets were labeled ex vivo with the nucleic dye, thiazole orange.^15^ We devised a gating strategy (Figure IV in the online-only Data Supplement) to determine the proportional usage of these newly formed platelets relative to older nonreticulated platelets^16^ during aggregate formation. Using PRP from untreated healthy volunteers stimulated by AA (1 mmol/L) or ADP (20 μmol/L) until 40% of platelets were aggregated the relative composition of the nonaggregated platelet population was assessed by flow cytometry. After aggregation, there was a significant reduction in the relative proportion of reticulated platelets indicating that reticulated platelets contributed overproportionately to aggregate formation (Figure 4A and 4B). Subsequent examination of formed aggregates by flow cytometric imaging confirmed the presence of reticulated platelets in the majority of aggregates (Figure 4C).

### Reticulated Platelets Undermine Platelet Inhibition by DAPT in Patients Taking Thienopyridines but Not in Patients Taking Ticagrelor

**Figure 5.**
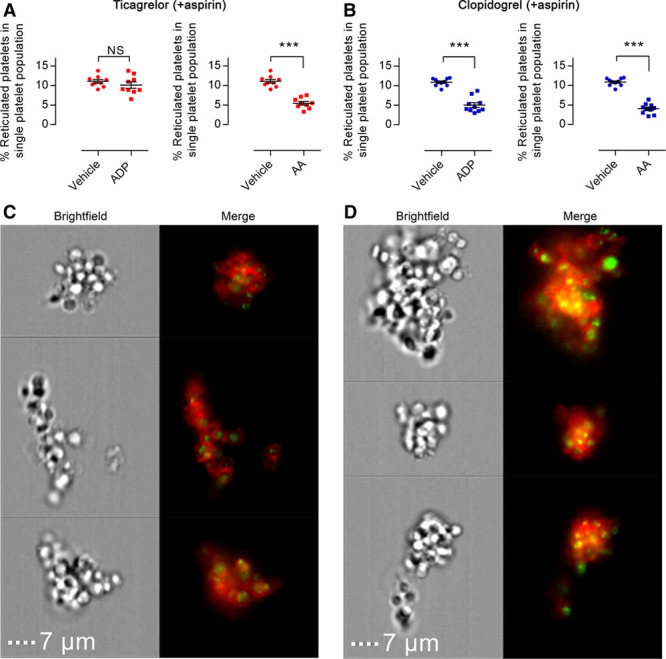
In patients the response of reticulated platelets to ADP is inhibited to a greater extent by ticagrelor than by clopidogrel. The reticulated platelet subpopulation among nonaggregated single platelets was assessed by flow cytometry in platelet-rich plasma incubated with vehicle, ADP, or arachidonic acid (AA). Samples were obtained from patients taking (**A**) ticagrelor or (**B**) clopidogrel (in addition to aspirin). Representative flow cytometric images of ADP-stimulated platelet aggregates (platelets red; mRNA green) formed in samples from patients taking aspirin plus (**C**) ticagrelor or (**D**) clopidogrel. Scale bars, 7 μm. Data presented as individual data points with overlaid mean±SEM and compared by paired *t* test (n=9–10; ***P<0.001).

Differential effects of thienopyridine- and ticagrelor-mediated inhibition on reticulated platelet populations were assessed in patients with established stable coronary artery disease who received DAPT comprising either aspirin+clopidogrel or aspirin+ticagrelor. Patients were assessed for pharmacological efficacy by testing of platelet reactivity using light transmission aggregometry. All patients had a final aggregation to ADP (20 μmol/L) of <43%, with those receiving ticagrelor considerably lower (Table I in the online-only Data Supplement). Thiazole orange stained PRP was incubated with vehicle, AA, or ADP for 5 minutes and reticulated proportion of the nonaggregated platelets assessed by flow cytometric imaging. In both the therapy groups, on stimulation by AA, reticulated platelets disappeared from the nonaggregated platelet population, reducing from 10.9±0.3% to 4.0±0.4% (*P*<0.001) in aspirin+clopidogrel patient samples and from 11.1±0.5% to 5.4±0.5% (*P*<0.001) in aspirin+ticagrelor patient samples (Figure 5A and 5B). This indicated an overproportional recruitment of reticulated platelets into the formed aggregates. In line with above data from AA-stimulated samples, stimulation by ADP of samples from patients receiving aspirin+clopidogrel caused a significant reduction in the relative population of reticulated platelets in the nonaggregated population, from 10.9±0.3% to 5.0±0.6% (Figure 5B; *P*<0.01). However, in contrast to these observations, stimulation with ADP of PRP from patients receiving aspirin+ticagrelor did not result in a drop of the reticulated platelet population (11.1±0.5% to 10.1±0.8%; Figure 5A, *P*>0.05). The absence of a change in proportion indicates a proportionally equivalent recruitment of reticulated and nonreticulated platelets to aggregates. Qualitative analysis of the imaged formed aggregates from each patient group demonstrated that in samples from patients receiving aspirin+ticagrelor, there were few reticulated platelets dispersed throughout the aggregate (Figure 5C), whereas in samples from patients receiving aspirin+prasugrel, there were increased numbers of reticulated platelets primarily located in the core of the formed aggregates (Figure 5D).

## Discussion

In our previous study, we demonstrated through in vitro modeling that drug-free platelets can act as seeds for aggregate formation during antiplatelet therapy.^10^ Here, we have studied the impact of platelet turnover, including the influences of reticulated platelets, during standard DAPT in both healthy volunteers and stable cardiovascular patients. Furthermore, we have compared thienopyridines with ticagrelor and from our results provide a potential pathophysiological mechanism that unites previous, but separate, associations between differential effectiveness of P2Y_12_ receptor inhibition, HTPR, immature platelet counts, and thrombotic risk.^3,6,13,14^ We directly demonstrate that after stimulation, reticulated platelets are overproportionately recruited to aggregates where they can act as seeds for larger aggregate formation and by interplay with drug pharmacokinetics provide a causative mechanism for observed HTPR.

Key to explaining our observations is an understanding that the formation of a drug-free, uninhibited, subpopulation of platelets during DAPT occurs as a result of platelet turnover and drug pharmacokinetics. In terms of standard therapy, aspirin is a short-lived but irreversible inhibitor of platelet cyclooxygenase-1. Similarly, the thienopyridines, prasugrel or clopidogrel acting through their active metabolites, are pharmacokinetically short-lived and are irreversible antagonists of platelet P2Y_12_ receptors. When used as DAPT, this combination of aspirin plus thienopyridine produces inhibition of circulating platelets. However, as we model in vitro and demonstrate ex vivo, neither aspirin nor prasugrel (or PAM) seem present in circulating blood at sufficient levels to inhibit the responses of exogenous platelets added in vitro. One potential explanation for this observation is that these drugs are present in effective inhibitory concentrations only within the portal circulation and so inhibit platelets as they pass through, as has been suggested for aspirin for >30 years.^17^ This would also explain why platelets newly released from the bone marrow are either poorly or not inhibited by aspirin and thienopyridines.^18,19^ One should not overlook, however, the alternative explanation that because of their short half-lives within the circulating blood, the active forms of thienopyridines may have insufficient time to interact with exogenously added platelets in our test system. In contrast, as expected, ticagrelor as a longer lasting (plasma half-life of ≈8 hours) direct acting reversible antagonist of P2Y_12_ receptors^12^ inhibited the responses to ADP of exogenously added platelets.

As well as modeling these interactions with regard to standard tests of platelet reactivity,^4^ our imaging techniques demonstrated that exogenously added uninhibited platelets were clustered at the cores of aggregates formed in response to ADP in samples from volunteers receiving aspirin+clopidogrel, consistent with their ability to act as seeds for aggregate formation. As hypothesized previously,^12^ the longer half-life and reversible binding of ticagrelor, in contrast to the irreversible binding of prasugrel, means it is present and able to act on the exogenous drug-free, uninhibited, platelet subpopulation. It can be noted that ticagrelor might in addition act pleiotropically on adenosine uptake to influence platelet function,^20,21^ but we did not test this possibility. It was notable that the recovery of the response to AA caused by the addition of exogenous drug-free platelets was blunted in samples prepared from individuals receiving aspirin+ticagrelor compared with those receiving aspirin+prasugrel. This is consistent with P2Y_12_ blockade reducing the amplifying effects of thromboxane A2 produced in response to AA^22–26^ and confirmed our in vitro observation that circulating ticagrelor, unlike prasugrel, may provide additional compensation for the loss of cyclooxygenase-1 inhibition noted in individuals with elevated platelet turnover.^26,27^

In our in vitro and ex vivo models, we stained or labeled uninhibited platelets to allow determination of their function as a subpopulation. Examination of the definitive drug-free population in patients is less straightforward. Newly formed immature platelets are also called reticulated platelets because of the presence of residual cytosolic mRNA. Dyes such as thiazole orange, which stain nucleic acids, are routinely used for determining the percentage of reticulocytes (including platelets) in blood samples.^28^ Accepting that the emergence of a drug-free platelet subpopulation occurs as a result of platelet turnover and the associated release of newly formed platelets, analyses of newly formed platelets in samples can be used to inform on the behavior of drug-free platelets. We have demonstrated under our particular conditions that thiazole orange staining of PRP identifies those platelets with the highest mRNA content. We therefore utilized this approach to track newly formed platelets during aggregate formation. In samples from healthy volunteers not taking antiplatelet drugs, reticulated platelets were overproportionally recruited to the formation of aggregates, indicating that under normal physiological conditions, they are important drivers of the aggregation process. This finding concurs with previous reports by ourselves, and others, that newly formed reticulated platelets possess inherently greater reactivity and have a greater propensity for recruitment to thrombi.^29^

Finally, we sought to determine whether such a mechanism was also present in patient samples. We recruited patients with established, stable coronary artery disease taking clopidogrel or ticagrelor plus aspirin and confirmed drug efficacy to ensure that patients exhibiting HTPR were not included in our analyses. As in our in vitro and ex vivo modeling, the behavior of reticulated platelets matched that of uninhibited platelets. In samples taken from patients receiving aspirin+clopidogrel, there was a significant loss in the proportion of reticulated platelets from the nonaggregated single platelet population after ADP stimulation, whereas strikingly, in patients receiving aspirin and ticagrelor, no proportional change was observed. Similarly, examination of the formed aggregates revealed that in response to ADP, reticulated platelets were clustered in the core and present in greater proportion in samples from patients receiving clopidogrel+aspirin than in samples from patients receiving ticagrelor+aspirin.

Our results support a mechanism through which newly formed uninhibited reticulated platelets play a key role in the limiting the effectiveness of particular antiplatelet therapies. Furthermore, we provide functional evidence and unique images substantiating the observed impact of subpopulations of reticulated drug-free platelets on the formation of platelet aggregates under recommended clinical testing settings.^30,31^ Together, these are consistent with emerging evidence establishing a link between reticulated platelets and platelet responsiveness to short-lived P2Y_12_ antagonist (thienopyridines)^11,32^ but not ticagrelor.^33^ Although there have been recent indications of similar short-term efficacy for prasugrel and ticagrelor in patients with acute myocardial infarction,^34^ no comparisons or subgroup analyses have yet been conducted in conditions of high platelet turnover. Moreover, our data suggest that in vivo the use of ticagrelor rather than clopidogrel or prasugrel may mitigate incomplete inhibition of thromboxane A2 formation by prophylactic aspirin,^4^ consistent with our previous reports on the importance of P2Y_12_ receptors in amplifying responses to platelet produced thromboxane A2.^35–38^

In conclusion, we demonstrate a functional mechanism for newly formed reticulated platelets to drive thrombus formation even during standard DAPT. Furthermore, our study demonstrates that ticagrelor may be more efficacious than thienopyridine (prasugrel or clopidogrel) therapy for mitigating HTPR associated with the generation of new platelets during standard antithrombotic regimens. In turn, this illustrates the importance of considering platelet turnover and the pharmacological inhibition of the reticulated platelet subpopulation in attaining optimal antithrombotic potential. Finally, given the central role for platelet turnover in our model, patients with conditions such as diabetes mellitus and chronic kidney disease, where increased platelet turnover has been identified or suspected, may particularly benefit from such considerations.

## Acknowledgments

We are grateful to Professor Sussan Nourshargh for use of confocal microscopes.

## Sources of Funding

This study was supported by grants from the Medical Research Council, the British Heart Foundation (PG/12/68/29779, PG/15/47/31591, and FS/12/53/29643), Wellcome Trust (101604/Z/13/Z), AstraZeneca, and the William Harvey Research Foundation.

## Disclosures

T.D. Warner has received research grant funding and consultancy fees from Astra Zeneca. The other authors report no conflicts.

## Supplementary Material

## Highlights

- We demonstrate a functional mechanism by which newly formed reticulated platelets can drive thrombus formation even during standard dual-antiplatelet therapy.
- Study reveals underlying reasons to consider platelet turnover and drug pharmacokinetics in the selection of appropriate antiplatelet therapies for optimal antithrombotic protection.
