# Supplementary material for: Newly Formed Reticulated Platelets Undermine Pharmacokinetically Short-Lived Antiplatelet Therapies
Source: Arterioscler Thromb Vasc Biol. 2017 Mar 2;37(5):949–56. doi: 10.1161/ATVBAHA.116.308763 (PMC5405774; doi:10.1161/ATVBAHA.116.308763)
Supplement: Supplementary file 1 [file atv-37-949-s001.pdf]

Methods and Materials

Ethics statement

All studies were conducted according to the principles of the Declaration of Helsinki and approved by St Thomas's Hospital Research Ethics Committee (Ref. 07/Q0702/24). All volunteers gave written informed consent before entering the study.

Study participants

Male, Caucasian volunteers aged 18-40 years were recruited and participated in the study. Health status was determined through medical history and physical examination, including blood pressure, pulse rate, blood chemistry and urinalysis. Volunteers with normal clinical profiles were included in the study. Where appropriate age and sex matched controls were also recruited for the donation of drug-free platelet populations. Patients with established stable coronary artery disease, aged 33-80 receiving aspirin and a $P2Y_{12}$ receptor antagonist following acute coronary syndrome in the last 12 months were recruited for study. The exclusion criteria included diabetes, current smoker, acute myocardial infarction (within 1 month), bleeding diathesis, and concomitant use of other anti-platelet drugs (e.g. cilostazol), non-steroidal anti-inflammatory drugs (NSAIDs) or anti-coagulants. Samples were obtained at least 4-6 hours since last dose and compliance was assessed by light transmission aggregometry (LTA).

Ex-vivo healthy volunteer study protocol

Prior to commencing with study protocol (Supplemental Figure 1), all healthy volunteers had abstained from aspirin, NSAIDs and any other anti-platelet therapy for 14 days. The volunteers were divided into two groups of 10 and received DAPT comprising aspirin once daily (75 mg; Nu-Seals Cardio 75, Alliance Pharmaceuticals Ltd, Chippenham, UK) plus either prasugrel (10 mg o.d.; Effient®, Eli Lilly, RA Houten, The Netherlands) or ticagrelor (90 mg b.i.d.; Brilique®, AstraZeneca, Södertälje, Sweden) for 14 days. Compliance was assessed by interview. Blood samples were collected prior to and on completion of therapy at both plasma peak level (30 min after the last prasugrel dose, 2 h after the last ticagrelor dose) and 6 h after the last dose.

Blood collection and isolation of platelets

Blood was obtained by venepuncture from the median cubital vein using a 19G butterfly needle into tri-sodium citrate (0.32% w/v final; Sigma, Poole, Dorset, UK). Blood from healthy volunteers free from antiplatelet drugs was centrifuged at 175 *x g* for 15 min to obtain platelet-rich-plasma (PRP). Where appropriate platelets were isolated from PRP by further centrifugation (750 *x g*, 10 min) in the presence of prostacyclin (PGI_2, 1 µg/ml; Tocris, UK) and apyrase (0.02 U/ml; Sigma Aldrich, UK). The resulting pellet was washed in modified Tyrode's (MTH) buffer (containing 134 mmol/L NaCl, 2.9 mmol/L KCl, 0.34 mmol/L Na_2HPO_4, 12 mmol/L $NaHCO_3$ and 1 mmol/L $MgCl_2$; pH 7.4) containing HEPES (20 mmol/L; Sigma, UK) and 0.02 U/ml apyrase (Sigma Aldrich, UK) and re-suspended in an equal volume of modified Tyrode's buffer.

Platelet labelling

Platelet suspensions were centrifuged (750 *x g*, 10 min) in the presence of PGI_2 (1

µg/ml) and resuspended in Diluent C (Sigma Aldrich, UK) containing 2 µmol/L PKH26 or PKH67 (Sigma Aldrich, UK). Following 5 min incubation, BSA-containing MTH buffer was added and samples were centrifuged (750 *x g*, 10 min) again in the presence of PGI_2 (1 µg/ml). Alternatively, platelet suspensions were incubated with anti-human CD61 antibody conjugated to APC (1:50 final dilution; clone VI-PL2, eBioscience, Hatfield, UK). Where appropriate, labelled platelets were re-suspended in either modified Tyrode's buffer (containing 0.35% bovine serum albumin) to $3x10^8$ platelets/ml or platelet poor plasma (PPP), containing 0.1 U/ml apyrase, obtained by centrifugation (15000 *x g*, 2 min). To identify reticulated platelet population, PRP was incubated with thiazole orange (200 ng/ml final; Sigma, UK) for 30 minutes at room temperature.

***In vitro* treatment of platelets**
Citrated blood or PRP was incubated with aspirin (30 µmol/L), prasugrel active metabolite (3 µmol/L, PAM), ticagrelor (1.35 µmol/L) or vehicle for four hours or 30 minutes respectively at 37°C. Concentrations of each treatment were chosen to reflect expected plasma concentrations.[1-3] Platelet suspensions containing different treatments were combined in various proportions and incubated for 20 minutes at 37°C. For in vitro modelling, post mixing plasma levels were adjusted to reflect mid-dose drug levels (PAM: 0 mol/L; ticagrelor: 1.35 µmol/L) before testing by LTA.

Light transmission aggregometry
Aggregation in response to 5 and 20 µM ADP or arachidonic acid (AA; 1 mmol/L; Sigma, UK), was measured in a Bio/Data PAP-8E turbidometric aggregometer using the principle of LTA. Maximal and final percent aggregation values after five minutes were recorded. When studying reticulated platelets, PRP was stimulated for 5 minutes or until it reached 40% aggregation where appropriate. To ensure patients demonstrating HTPR were not included, LTA was also used to assess therapy efficacy in patient populations using the maximal aggregation cutoff values AA 1 mmol/L <20%, and ADP 20 µmol/L <59%.[4-6]

Flow cytometric imaging
Labelled platelet samples obtained by LTA were acquired on an ImageStream^X MarkII imaging flow cytometer (Amnis Corporation, WA, USA) which combines the quantitative power of flow cytometry with high content image analysis by acquiring up to twelve images simultaneously of each cell or object including brightfield, scatter, and multiple fluorescent images. Ideas software (Amnis Corporation, WA, USA) was used for post-acquisition analysis. Briefly, single platelet populations were gated by size and CD61-Alexa647 positivity of non-stimulated samples and confirmed visually by acquired images. Aggregates were size, and PKH26+PKH67-positive events or CD61-Alexa647 positivity.

Confocal microscopy
Aggregates were fixed by addition of 1% paraformaldehyde (PFA), transferred to a microscope slide, mixed with hard set mounting medium (VECTASHIELD HardSet Mounting Medium, Vector Laboratories, UK) and covered with a coverslip. Platelet aggregates were then imaged using a Zeiss LSM 5 PASCAL confocal laser-scanning microscope incorporating a 63 x oil-dipping Plan-APOCHROMAT objective (numerical aperture 1.4 and resolution 0.28 µm). Z-stack images were captured using the multiple track-scanning mode. Z-stacks obtained by confocal microscopy

were processed with IMARIS (Bitplane AG, Switzerland) by rendering surfaces on captured fluorescence.

Data presentation

Graphs and statistical analysis were generated using GraphPad Prism 6 software (GraphPad Software Inc. USA). Data were expressed as mean±SEM and all statistics were generated using a one-way or two-way ANOVA, with a Tukey's post-test or column statistics with a one sample t-test, as appropriate. Significance was defined as $p=<0.05$.

References:

1. Wallentin L, Varenhorst C, James S, Erlinge D, Braun OO, Jakubowski JA, Sugidachi A, Winters KJ, Siegbahn A. Prasugrel achieves greater and faster p2y12receptor-mediated platelet inhibition than clopidogrel due to more efficient generation of its active metabolite in aspirin-treated patients with coronary artery disease. *Eur Heart J*. 2008;29:21-30
2. Storey RF, Husted S, Harrington RA, Heptinstall S, Wilcox RG, Peters G, Wickens M, Emanuelsson H, Gurbel P, Grande P, Cannon CP. Inhibition of platelet aggregation by azd6140, a reversible oral p2y12 receptor antagonist, compared with clopidogrel in patients with acute coronary syndromes. *J Am Coll Cardiol*. 2007;50:1852-1856
3. Nylander S, Schulz R. Effects of p2y12 receptor antagonists beyond platelet inhibition - comparison of ticagrelor with thienopyridines. *Br J Pharmacol*. 2016;173:1163-1178
4. Gurbel PA, Antonino MJ, Bliden KP, Dichiara J, Suarez TA, Singla A, Tantry US. Platelet reactivity to adenosine diphosphate and long-term ischemic event occurrence following percutaneous coronary intervention: A potential antiplatelet therapeutic target. *Platelets*. 2008;19:595-604
5. Gurbel PA, Bliden KP, Butler K, et al. Randomized double-blind assessment of the onset and offset of the antiplatelet effects of ticagrelor versus clopidogrel in patients with stable coronary artery disease: The onset/offset study. *Circulation*. 2009;120:2577-2585
6. Gurbel PA, Bliden KP, DiChiara J, Newcomer J, Weng W, Neerchal NK, Gesheff T, Chaganti SK, Etherington A, Tantry US. Evaluation of dose-related effects of aspirin on platelet function: Results from the aspirin-induced platelet effect (aspect) study. *Circulation*. 2007;115:3156-3164
