# Supplementary material for: Newly Formed Reticulated Platelets Undermine Pharmacokinetically Short-Lived Antiplatelet Therapies
Source: Arterioscler Thromb Vasc Biol. 2017 Mar 2;37(5):949–56. doi: 10.1161/ATVBAHA.116.308763 (PMC5405774; doi:10.1161/ATVBAHA.116.308763)
Supplement: Supplementary file 2 [file atv-37-949-s002.pdf]

SUPPLEMENTAL MATERIAL

Armstrong: Reticulated platelets and the formation of thrombi

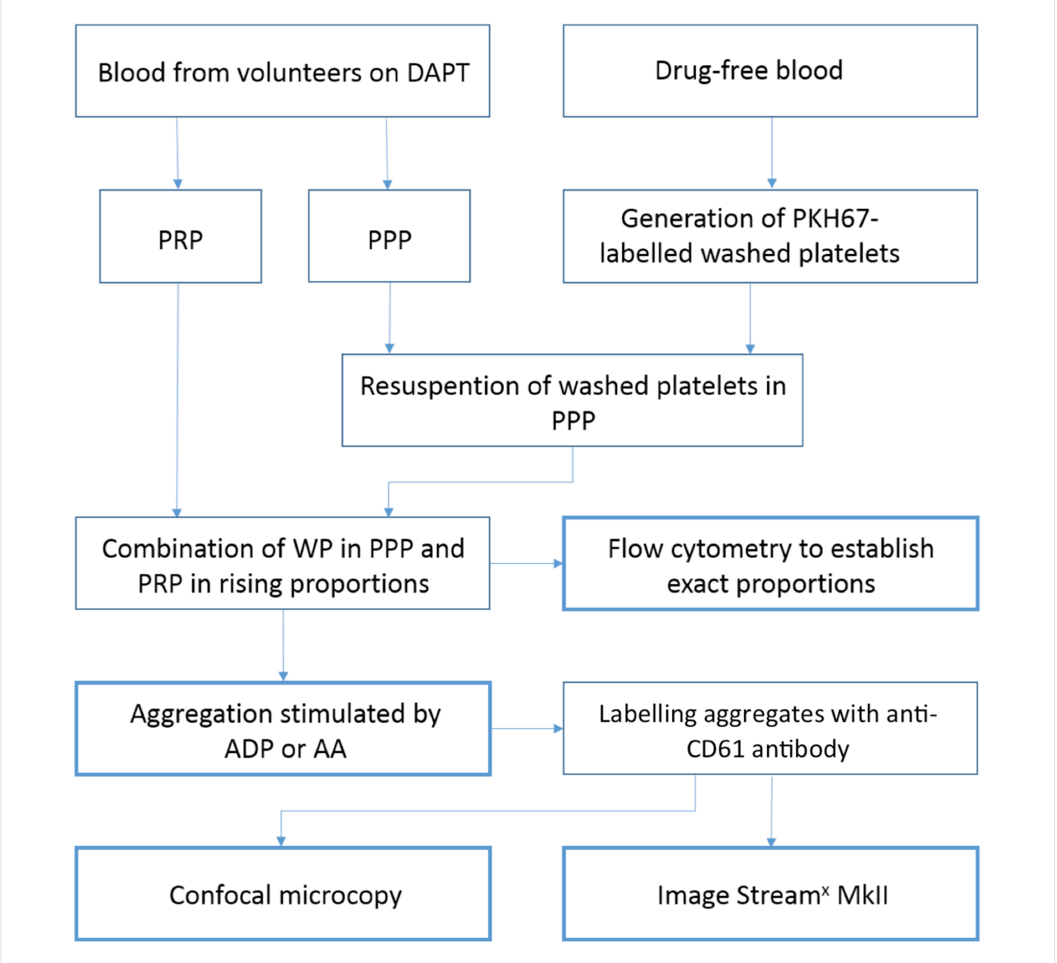

Supplemental Figure I: Flow diagram of study protocol for ex vivo healthy volunteer platelet sample testing.

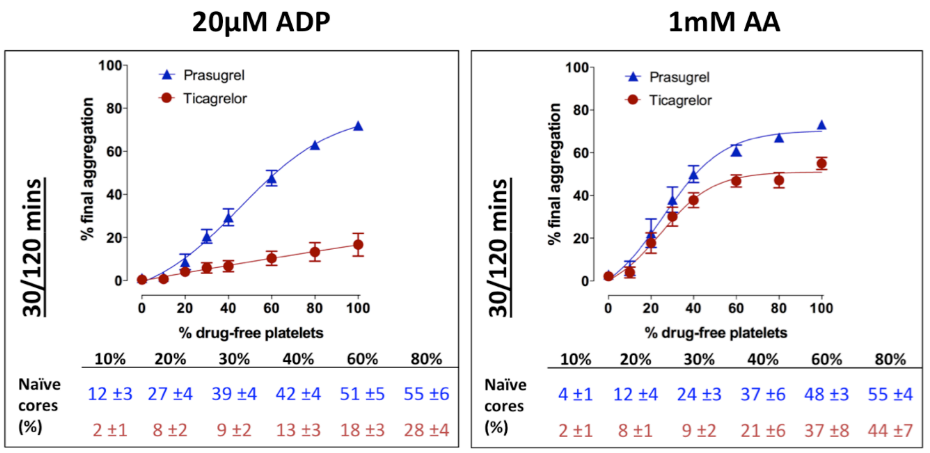

Supplemental Figure II: Drug-free platelets restore *ex vivo* aggregation responses differentially in presence of prasugrel or ticagrelor. PRP isolated from individuals receiving aspirin+prasugrel or aspirin+ticagrelor 30mins and 2hours respectively after last dose was mixed with increasing proportions of drug-free platelets and stimulated with (A) ADP 20µM or (B) arachidonic acid 1mM. (Inset tables) drug-free platelet cores (%) blind scored and calculated from flow cytometric images of platelet aggregates from corresponding samples. 104 aggregates assessed per individual sample, with data presented as mean±SEM and compared by two-way ANOVA (n=10).

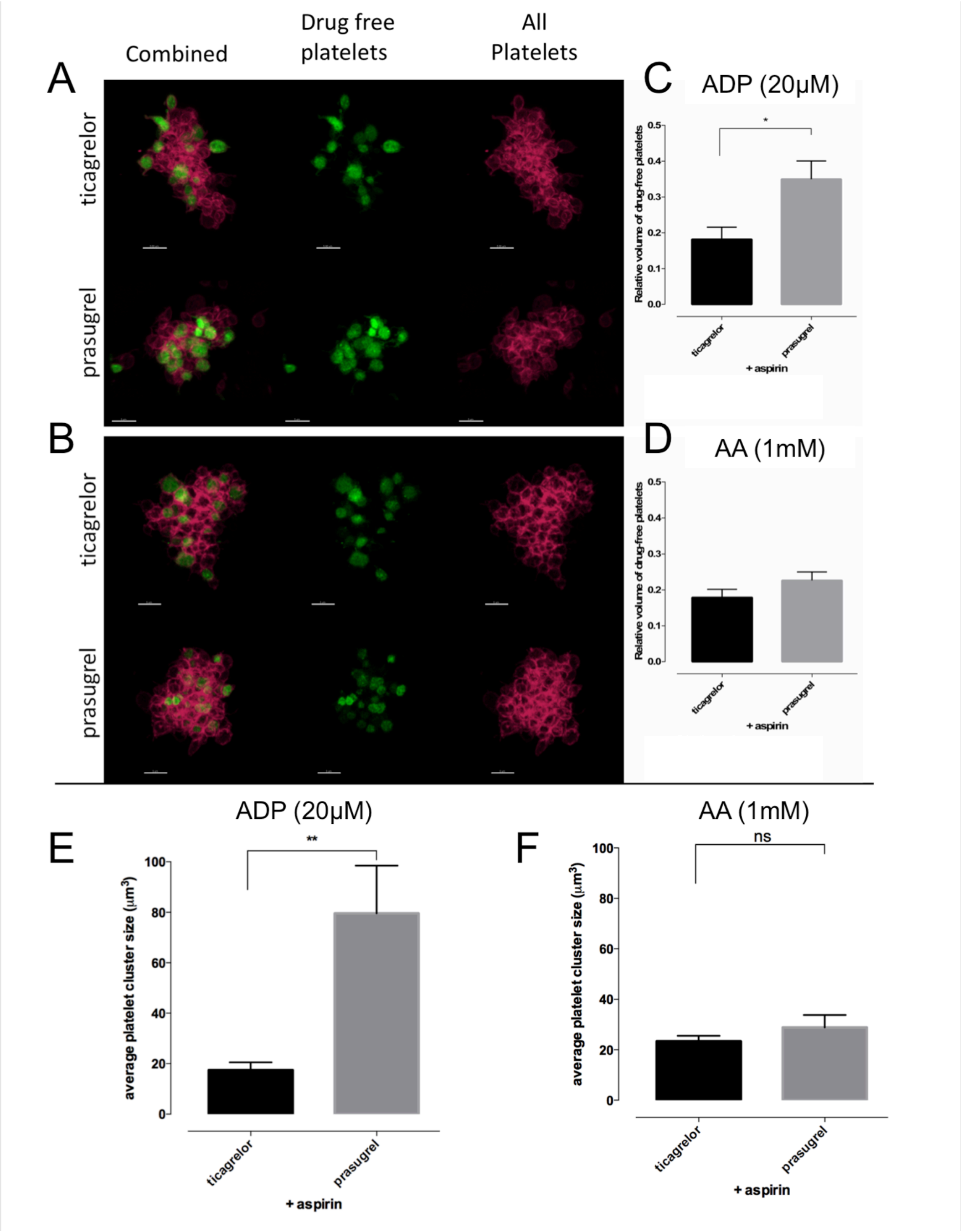

Supplemental Figure III: Drug-free platelets form cores within aggregates in presence of prasugrel but not ticagrelor. Representative confocal images of (A) ADP-stimulated or (B) AA-stimulated aggregates formed from 80%:20% mixtures of aspirin+prasugrel-inhibited PRP or aspirin+ticagrelor-inhibited PRP obtained 30mins and 2hours respectively after the last drug dose was administered (red) and drug-free platelets (green), conditions as in Figure 1. Images were analysed for (C, D) volume of the drug free platelet particles relative to the total aggregate volume and (E, F) average size of drug-free platelet clusters. Scale bars represent 5 μm. Data is presented as mean±SEM and compared by t-test (n=7-10; ***p<0.001).

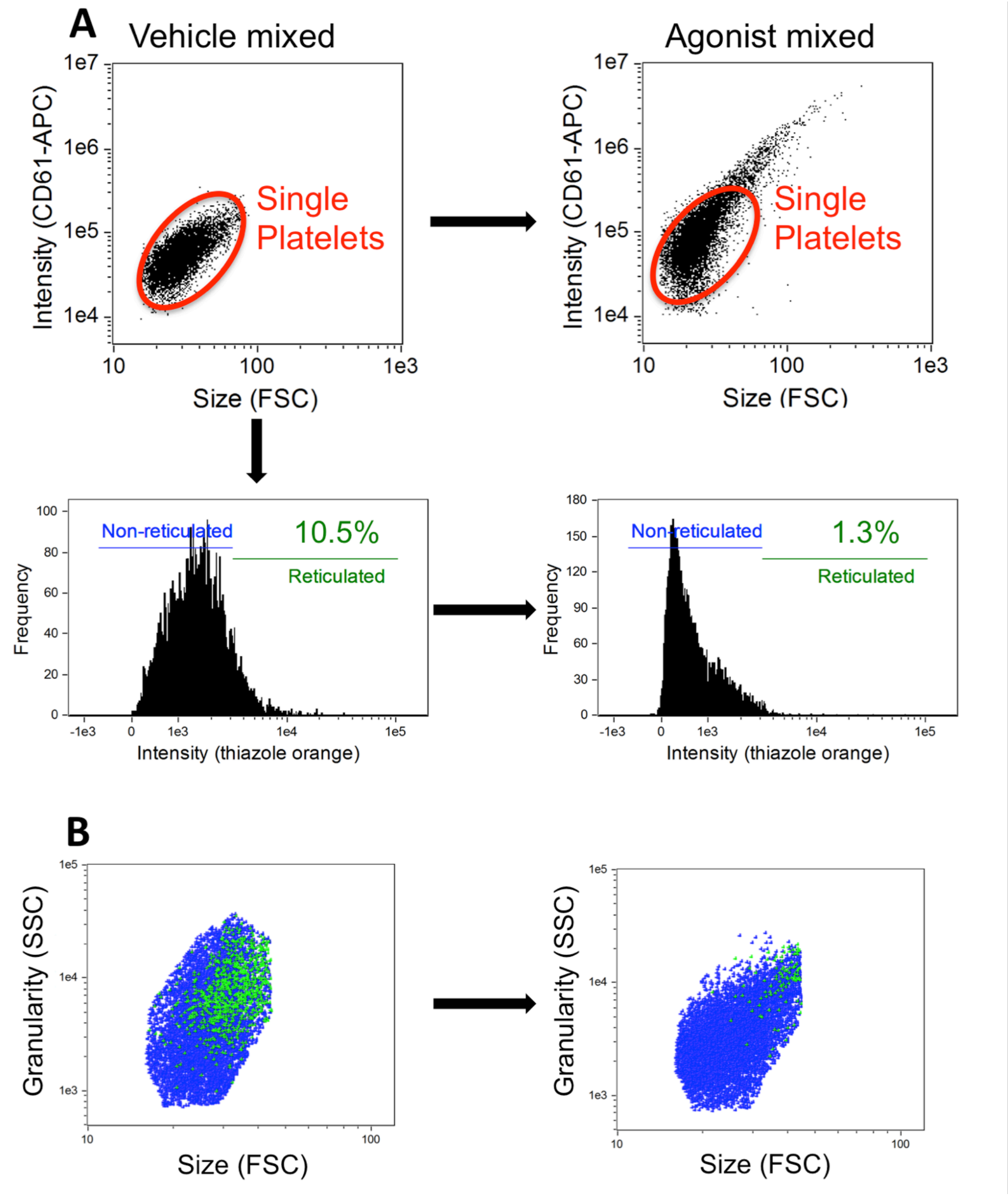

Supplemental Figure IV: Gating strategy to determine the proportional loss of newly formed platelets relative to older non-reticulated platelets during aggregate formation. (A) Single non-aggregated platelets in PRP identified by size (FSC) and CD61 positivity of vehicle mixed sample (left). Thiazole orange histogram of identified single platelets was then used to identify range incorporating brightest 10% (designated 'reticulated'). Using both these parameters, agonist mixed (right) samples were analysed and % 'reticulated' calculated. (B) Representative size (FSC) and granularity (SSC) of identified populations in vehicle mixed (left) and agonist mixed (right) samples.

	Clopidogrel (+ aspirin)			Ticagrelor (+aspirin)		
**Max Aggregation**	**Mean**	**SD**	**N**	**Mean**	**SD**	**N**
AA 1mM	1.4	1.0	10	4.8	6.3	9
ADP 20µM	41.5	10.8	10	26.2	13.2	9
**Final Aggregation**	**Mean**	**SD**	**N**	**Mean**	**SD**	**N**
AA 1mM	0.0	0.0	10	3.3	6.7	9
ADP 20µM	26.1	13.9	10	12.9	11.3	9

Supplemental Table I: **LTA values for stable CVD patients.**
