# Supplementary figures and images for: Newly Formed Reticulated Platelets Undermine Pharmacokinetically Short-Lived Antiplatelet Therapies
Source: Arterioscler Thromb Vasc Biol. 2017 Mar 2;37(5):949–56. doi: 10.1161/ATVBAHA.116.308763 (PMC5405774; doi:10.1161/ATVBAHA.116.308763)

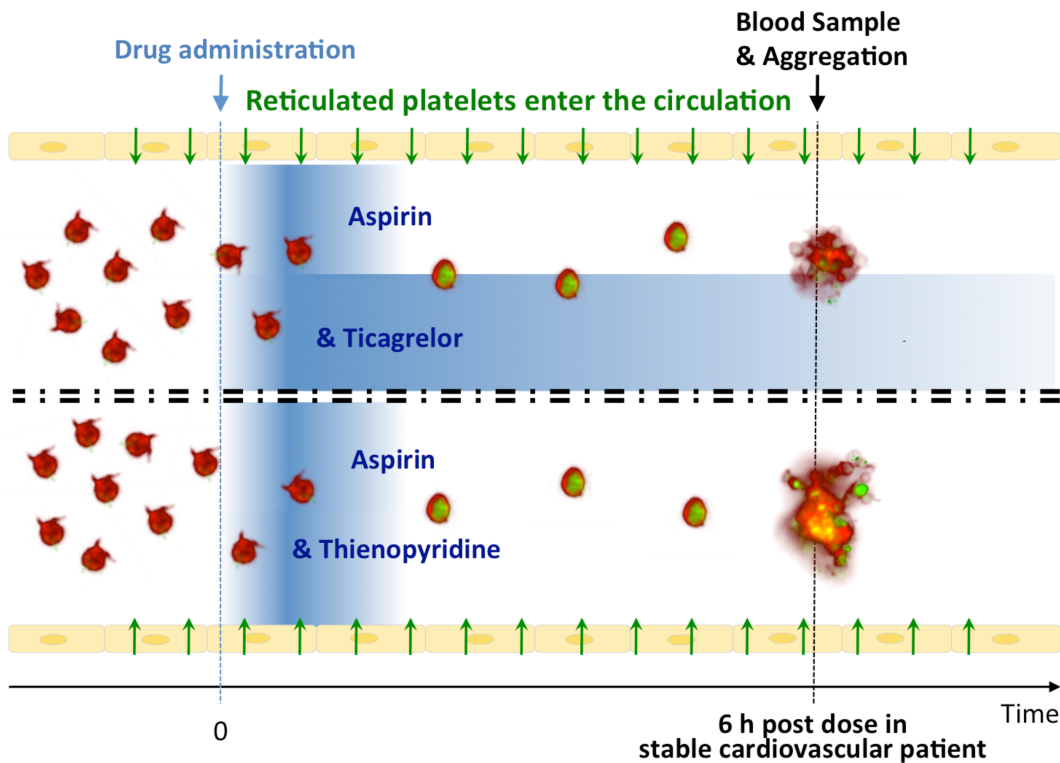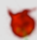

Non-reticulated platelet

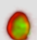

Reticulated platelet

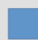

Active drug

Supplement: Supplementary file 3 [file atv-37-949-s003.pdf]
